# Even an Activated Long-Term Memory System Still Needs a Separate Short-Term Store: A Reply to Cowan (2019)

**DOI:** 10.1037/bul0000204

**Published:** 2019-08

**Authors:** Dennis Norris

**Affiliations:** 1 MRC Cognition and Brain Sciences Unit, University of Cambridge

**Keywords:** working memory, short-term memory, STM, long-term memory, LTM

## Abstract

In Norris (2017), I explained why the notion of activated LTM (long-term memory) combined with a focus of attention was unable to perform the computations required to support short-term memory (STM) and argued that those extra computations must require a separate STM system. Cowan (2019) made the alternative proposal that this full set of computations is better conceptualized as a unitary system of activated LTM. To this he added a pointer system, the ability to perform variable binding, and an unspecified model of STM that acts as a front end to LTM. This appears to be simply an exercise in relabeling. Furthermore, without a computational specification of how the components work, the model lacks the ability to simulate even the most basic STM phenomena. If the model were specified in more detail it seems almost inevitable that it would contain something instantly recognizable as an STM system.

When I quoted Cowan and Chen (2008) as saying that “although the mechanisms of short-term memory are separate from those of long-term memory, they are closely related” (their p. 104), I thought Neslon Cowan and I had little to disagree about. I also consider Cowan’s (1988, 1999) idea of a focus of attention (FoA) to be a useful way of thinking about control processes in working memory. So why is it that Cowan (2019) now feels the need to respond to the arguments I presented in Norris (2017)? The framework he presents builds on Cowan (1999) and Cowan (1988), and he now argues that there is no separation between short-term and long-term memory systems after all. He does this by endowing both the FoA and aLTM (activated LTM) with additional properties and an unspecified model of STM. In order to dispense with a separate STM he then deems all of this to be a unitary LTM system. Cowan has not shown that there is no need for a separate STM system, he has just redefined it out of existence.

Norris (2017) highlighted the inadequacy of models that embody the claim that STM can be supported entirely by aLTM. In that article, I explained that the computational requirements of storing information over the short-term entail additional mechanisms to activation. In particular, memory must be able to support the construction of novel representations that have no preexisting representation in LTM. At the very least, this requires the ability to store multiple tokens of a given type (“the problem of two”) and to perform variable binding. Any LTM system must be supplemented by extra mechanisms that are required to store information over the short term. Consider the need to store multiple tokens of a given type. I gave the example of the easily remembered sentence, “Buffalo buffalo buffalo buffalo buffalo” and argued that it was implausible to assume that LTM stored five copies of the phonological word form /bʌfələʊ/, just in case one was ever asked to repeat this sentence. The binding problem can be exemplified by the sentence, “The young boy saw the boy who was singing.” As I noted in my original article (Norris, 2017),

Here the problem is not simply representing the order of the words, but appreciating that there are two different boys, one of whom is singing and one of whom is young. It is necessary to represent both multiple tokens and the bindings between each of those tokens and other components of the sentence. (p. 1000)

Similar problems arise in the case of visual STM, where memory for an array of random dots of the same size requires binding multiple tokens of dots to their locations.

Cowan (2019) agreed that the simple notion of STM as activated LTM cannot solve these problems. But even in the abstract of his commentary, he conceded the need for separate short-term storage system when he stated that “models of STM storage can serve as the front end of an LTM learning system rather than being separate” (Cowan, 2019, p. 822). The point is reinforced later when he stated that

[t]he viability of an approach involving aLTM with new learning does not depend on coming up with a separate serial order memory model specifically within the embedded-processes framework, inasmuch an adequate model of serial order memory in STM formulated by another investigator also could also serve as the long-term learning mechanism. (p. 832)

Cowan (2019) recognized the need for a model of STM, but instead of considering it a separate STM system, he instead tried to take an existing model of STM and call it a front end to aLTM. In Table 1 he states “-STM-copy theories might be reclassified as the front end of long-term learning”. This is simply an exercise in relabeling.^1^

Even though Cowan’s acceptance of the need to incorporate a model of STM has undermined his own case for calling his theory aLTM, it is still worth taking a closer look at the theory to examine how the various components work together and how they relate to standard two-store models. First, it should be noted that for more than 25 years there has been an expectation that new theories of STM should be presented as computational models (Burgess & Hitch, 1992; Henson, 1998; Page & Norris, 1998, for review see Hurlstone, Hitch, & Baddeley, 2014). This has at least two advantages. First, we can run simulations to assure ourselves that the model really can simulate the target data set. Second, it doesn’t matter too much if we cannot pigeonhole the model into categories such as, for example, “two-store,” “one store,” or “uses activation.” We can see how it works, and then, should we wish to do so, assign it an informative label. Cowan reversed this process: He started with a label and then asserted that there might exist a model that could be labeled in this way. If the final step were to construct a computational model that fit the label and simulated the data, I would have few complaints. I might have been tempted to question whether the label seemed appropriate, but at least I would have been convinced that the model could work, which is the important thing. Could there be a model that works according to the principles Cowan has espoused? Given that the “model” is expressed only verbally, we cannot be sure.

Cowan’s unitary model has five core features, aLTM, FoA, a pointer system, rapid learning, and a model of STM. With these extra features, aLTM is now assumed to be able to perform variable binding, to instantiate multiple tokens, and to create temporary representations that can support performance in STM tasks. The extras are there to fulfill the function of an STM system that performs computational functions that are distinct from those of LTM. They allow Cowan to smuggle STM into LTM.

## Activation

The core feature of aLTM is activation. In Norris (2017), I suggested that “it seems reasonable to ask what computational function is performed by activation that enables it to encode, maintain, and retrieve information from STM” (p. 998). Cowan (2019) replied that “[a]ctivation, then, is simply the degree of availability for retrieval” (p. 834). But this doesn’t answer the question about the computational function served by activation. What it says is that given some behavior (retrieval), we can infer that LTM is in some underlying state called *activation*—but all we know about that state of activation is that it is something that caused the behavior that we used to infer that activation in the first place. We’re no wiser about the computational role of activation. The absence of any clear computational definition of activation is apparent in Cowan’s concluding sentence, which reads as follows: “The exact meaning of activation and of the two alternatives may change as the pursuit to test them continues; changing definitions is a legitimate part of the progression of a science” (p. 842). In other words, we can always use the term activation, because we can always change what it means.

## The FoA and Pointers to aLTM

Cowan (2019) wrote the following:

The information held with the FoA could be described as a structured set of pointers, it would also serve as a portal to LTM learning. For example, to learn the list of digits *739482*, the individual might memorize *739*, then *48*, and then the association between these segments as *739–48*, subsequently incorporating the last digit to encode *739–48–2*. That reiterative process . . . would presumably be available for immediate recall. (p. 829)

I take this to mean that pointers do the job of representing sequences. This gives the FoA all of the computational power needed by an STM system, but Cowan still declined to call it STM. Given that aLTM does not have a representation of *979482* to begin with, the focus of attention must be focusing on something other than a subset of aLTM. The only other thing available is the representation stored in an STM system.

Norris (2017) discussed the issue of how to best label a system that relies on pointers:

If there is a system where the short-term store (STS) contains pointers to LTM, should we really call this an STS, or is it just a pointer system? My own inclination is to stick with the term STS, as the pointers are doing all of the hard work. (p. 1003)

But Cowan went beyond having a simple set of pointers and proposed that “a pointer system is expected in which a structured set of references to information in aLTM would be established . . . [and where] a set of items is apprehended with the FoA and then off-loaded into new LTM representations” (p. 838). Here, the hard work in not being done by pointers, but by a system labeled FoA which can construct structured representations and offload them to LTM. FoA has been allowed to subsume the processes normally considered to be part of a separate STM system. My preference remains to call that an STM system.

## The Role of Rapid Learning

Cowan proposed that some of the problems with aLTM can be overcome by invoking rapid learning and assumes that

information can be learned quite quickly, so newly learned structures (such as the serial positions of list items, spatial positions of array items, or binding of items to semantic roles) is processed by the FoA and is concurrently learned, resulting in new aLTM material that can be used on the trial (though learning may be imperfect, and later retrieval depends on interference and on retrieval cues). (p. 826)

However, increasing the speed of learning does not help aLTM escape its predicament. Rapid learning relies entirely on representations constructed by the FoA, but these must be different from the representations in aLTM, otherwise there would be no need for rapid learning. The FoA has now been given all of the power and storage capabilities of a separate STM system. If you can rapidly learn *979482* you have already managed to solve the problem of two (there are two *9*s). Cowan and I agree that this cannot be done with aLTM alone.

## A Model of STM as the Front end of LTM

When Cowan suggests that a model of STM might form the front end of LTM learning it is not clear whether he has a particular model of STM in mind. It is also unclear what STM can do that is beyond the capabilities of the newly endowed FoA. The model he devotes most space to discussing is Burgess and Hitch (2006). Like all connectionist models of STM, their model has multiple components with separate interacting layers of nodes. The layers perform the task of representing the specific sequence of items or events and transferring those temporarily constructed representations into LTM. Much the same happens in the model of Page and Norris (2009). As with all computational models of STM, there is a lot of weighty structure here. Cowan argued that the entirety of this mechanism can be reclassified as aLTM or FoA.

## Conclusion

The conclusion of this response remains the same as that of Norris (2017): A simple activation process would be unable to solve the “problem of two” or to store novel representations. Thus, it follows that any model that places an emphasis on storage by activated LTM must be supplemented by some additional mechanism that can represent multiple tokens and serial order. That additional mechanism must be able to perform the variable-binding operation required to construct novel representations and would then amount to what has been conventionally thought of as a *short-term store*. In fact, the resulting model would look very much like existing computational models of STM. Some might still prefer to describe this by saying that STM is aLTM. If it is made clear that there must be some additional mechanism and how that mechanism operates, at least we would know what they mean.

Cowan (2019) admitted that there must be some additional mechanism, but with only a verbal description to go on, it is far from clear what he meant or even whether his proposals would actually work. It seems likely that if his proposals were incorporated into an explicit computational model, they would work only to the extent that they instantiated the mechanism of some existing model of STM. This is apparent in the claim that “separate STM copy theories might be reclassified as the front end of long-term learning” (p. 824). In other words, you need a separate STM system. STM and LTM are still different, unless you “pretend” otherwise.

## Figures and Tables

**Table 1:** Responses to the Arguments for a Separate Copy of Information in STM

Description of argument	Cowan’s (2019) response against separate copy	My response to Cowan (2019)
1. Storage of new configurations is needed in STM	Everyone recognizes that there must be new, rapid learning of information in STM tasks (e.g., Keppel & Underwood, 1962), and the newly learned information is typically still in an activated state, aLTM, at the time of test (Cowan, 1999).	Few could disagree with the first part of this response, but it fails to address the question posed. The original question concerned the need to store novel representations that had no preexisting representation in LTM. This cannot be achieved just by assuming that the learning is rapid. I also pointed out that there must be continual long-term learning. On first encounter with some new event there must be some long-term learning, otherwise every encounter would be the same as the first, and learning would never get underway.
2. Token representations cannot be represented in aLTM, only types	aLTM includes rapid learning of information, and therefore can include the same episodic information about tokens that one adds to LTM (Cowan, 1999; Nairne & Neath, 2001)	The case against aLTM applies regardless of the speed of aLTM. It needs more than go-faster stripes—it simply does not have the necessary representational capacity to do the job. Adding that extra capacity turns it into an STM system. What we need to know is how rapid learning works and exactly how it is supposed to solve the problem.
3. No extant model of STM performance based on aLTM	Including new learning as part of aLTM changes the need because separate STM copy theories might be reclassified as the front end of long-term learning. Many long-term learning models exist. A few models deal explicitly with aspects of aLTM and new learning (Anderson & Matessa, 1997; Cowan, Rouder, Blume, & Saults, 2012).	The need is as great as ever. There are no computational models of STM performance based simply on activated LTM. The models cited are not models of aLTM, and the models in Cowan et al. (2012) do not simulate any of the benchmark phenomena of STM. To resort to reclassifying models of STM as part of aLTM is to admit defeat.
4. STM recall differs from LTM recall in its properties	There is evidence that long-term learning with repetition heavily relies on item-item associations (Zaromb et al., 2006) not just item-position as implied by Cumming, Page, and Norris (2003). LTM with reduced interference looks more similar to STM (Dewar, Alber, Butler, Cowan, & Della Sala, 2012; Ecker, Brown, & Lewandowsky, 2015; Ecker, Tay, & Brown, 2015). Unlike the usual procedures, STM can use semantic information (Potter, 1993), and LTM can be made to use phonological cues when such cues are best-suited to the encoding context (Morris, Bransford, & Franks, 1977). Order retention suffers in dyslexia within both STM and LTM (Martinez Perez, Majerus, & Poncelet, 2013; Szmalec, Loncke, & Page, 2011).	Cumming, Page, and Norris (2003) was not cited in Norris (2017), and it is not clear how item-item versus item-position associations has any bearing on the issue. I did point out that that phonological confusions in STM only occur at short retention intervals, after which confusions are likely to be semantic
8. Variable binding must be encoded into STM	Patients with hippocampal damage and LTM deficiency also show a deficit in variable binding, in sentence comprehension requiring variable binding for pronoun assignment (Kurczek, Brown-Schmidt, & Duff, 2013)	The argument was that we must have some way of performing variable binding. aLTM fails to offer an account of how these computations might be performed. Given Cowan’s reluctance to accept the standard interpretation of neuropsychological evidence for a separation between STM and LTM, it is surprising to find him placing such weight on the neuropsychological evidence from a single study. In their abstract Kurczek et al. (2013) say *“*This finding suggests that the hippocampus plays a role in maintaining and integrating information even over a very short discourse history”. Even if the conclusion were that the hippocampus, and only the hippocampus, plays a role in binding, any further conclusion about the role of aLTM depends on the additional assumption that the hippocampus is exclusively involved in LTM and could not be construed as implementing any part of a separate STM process.
9. Neuropathological deficits distinguish STM from LTM	Specific deficits in STM performance could come from deficient processes specific to STM maintenance (e.g., rehearsal: Cowan, 1988; or other kinds of deficient coding: Cermak, 1997; Morey & Bieler, 2013; Morey, Rhodes, & Cowan, 2019; Ruchkin, Grafman, Cameron, & Berndt, 2003). Also, LTM procedure used have not closely matched STM procedures used.	It is always possibly to attribute damage to stores to damage to processes. One need only claim that there is one process for reading out information in the short term and one for the long term. The neuropsychological evidence has recently been the subject of a special issue of the journal *Cortex* (Papagno & Shallice, 2019). In particular, see Logie (2019) for a critique of Morey, Rhodes, and Cowan (2019). Interestingly, the main theme in that issue is not whether STM and LTM are separate, which was largely taken for granted. The papers focus on presenting evidence for further fractionation of STM and working memory into separate buffers.
10. Tasks are impure measures of either STM or LTM	LTM learning may make use of use the focus of attention once for subspan lists but reiteratively for supraspan lists (Rhodes & Cowan, 2018), and the reiterative process could be impaired.	The response doesn’t speak to the argument. Given that tasks are impure measures (Atkinson & Shiffrin, 1968), it is hard to design tasks that involve only STM or only LTM. That is, this is a statement about what follows from the assumption of separate stores.
11. Neuroimaging as a correlation fallacy	The scientific method seeks the most parsimonious and adequate theory that can accommodate all of the evidence, including correlations and causation. The neuroscientific evidence for the embedded-processes approach includes correlational neuroimaging-behavior correspondences (e.g., Chein & Fiez, 2010; Cowan, 2011; Cowan et al., 2011; Kalm & Norris, 2017; Lewis-Peacock, Drysdale, Oberauer, & Postle, 2012; Li, Christ, & Cowan, 2014; Majerus et al., 2016; Öztekin, McElree, Staresina, & Davachi, 2008) and causal TMS evidence (Postle et al., 2006; Rose et al., 2016).	Given that there are no pure measures, neuroimaging data that implicate brain regions purported to be involved in LTM in STM tasks, is simply correlational and is to be expected from the two-store view. Such data should therefore not be taken as evidence that regions assumed to be responsible for LTM are performing the STM task.
		The scientific method does indeed seek the most parsimonious and adequate theory. However, aLTM is not formulated with sufficient precision to know whether it can accommodate the evidence. The appropriate metric of parsimony is not simply a count of the number of stores that a theory claims to have. We also have to count the number of ad hoc assumptions. By adding extra assumption and an extra STM model, the aLTM seems far from parsimonious. It has the potential to explain almost anything. Embedded memory systems will be subject to the same computational constraints as any other STM system. Calling them aLTM is simply another exercise in relabelling.
*Note.* STM = short-term memory; aLTM = activated long-term memory; LTM = long-term memory.		

## Footnotes

1 Cowan (2019) presented 11 arguments for a separate copy of information in STM (see his Table 1). Table 1 in the current article presents my response to Cowan’s commentary, along with his description of the original argument. I have ignored Arguments 5 through 7, as I did not make them.
